# Microorganisms in the rumen and reticulum of buffalo (*Bubalus bubalis*) fed two different feeding systems

**DOI:** 10.1186/s13104-016-2046-y

**Published:** 2016-04-27

**Authors:** Raul Franzolin, André-Denis G. Wright

**Affiliations:** Faculdade de Zootecnia e Engenharia de Alimentos, Universidade de São Paulo, Av. Duque de Caxias Norte, 225, Pirassununga, SP 13630900 Brazil; School of Animal and Comparative Biomedical Sciences, College of Agriculture and Life Sciences, University of Arizona, Tucson, AZ 85721 USA

**Keywords:** Methanogenic archaea, Bacteria, Rumen protozoa

## Abstract

**Background:**

The community of microorganisms in the rumen and reticulum is influenced by feeding as well as the species and geographical distribution of ruminant animals. Bacteria, methanogenic archaea and ciliate protozoa existing in the rumen and reticulum were evaluated by real-time polymerase chain reaction and light microscopy in buffalo in two feeding systems, grazing and feedlot.

**Results:**

No significant differences were observed in the total concentrations of bacteria/mL and archaea between rumen and reticulum, and between pasture and feedlots, or interactions between variables. However, the largest density of bacteria and smallest density of archaea was observed in the rumen of grazing animals. The total ciliates protozoa community was higher in grazing buffalo than those in the feedlot on a concentrated diet. There were significant interactions between location in the gastrointestinal tract (rumen vs reticulum) and types of diets (grazing vs feedlot) in the composition of ciliates.

**Conclusions:**

Our data showed differences in the microbial community of the rumen and reticulum between grazing and feedlot feeding systems demonstrating relevant changes in the microorganism:host relationship existing on rumen–reticulum ecosystem.

## Background

Ruminal bacteria have much higher metabolic activity than larger microorganisms (protozoa and fungi), and are responsible for most of the digestive process in ruminants [1]. The microbial environment in the rumen-reticulum is quite complex and dynamic due to several factors including mainly the type of diet. The microbial community varies with the presence of bacteria (10^10^–10^11^/g), methanogenic archaea (10^7^–10^9^/g), ciliate protozoa (10^4^–10^6^/g), anaerobic fungi (10^3^–10^6^/g) and bacteriophage (10^9^–10^10^ particles/mL) [2].

It was previously thought that 300–400 different species of bacteria existed in the rumen, but now using modern techniques based on 16S rRNA gene sequence analysis, over 2000 species are thought to exist in the rumen and over 5000 species in the human gastrointestinal tract [3]. Real-time polymerase chain reaction (PCR) has been successfully used to quantify the microbial population in the rumen [4, 5]. Most of rumen-reticulum bacteria are associated with the use of fiber accounting for 77 % of the total microbial population. However, <97 % have been identified [6].

The methanogenic archaea have aroused interest among ruminal microbiologists, who are trying to improve efficiency of the fermentation process and reduce the environmental impacts caused by enteric methane emissions. *Methanobrevibacter* species have been found in high densities in the rumen of buffalo fed three different diets [7], while buffalo fed wheat straw had more *Methanomicrobium* spp. [8].

Knowledge of the microbial community is critical to development of specific strategies to increase the efficiency of production of ruminant meat and milk with energy saving and reduction of methane production [9].

The objective of this study was to evaluate the concentration of bacteria, methanogenic archaea, and ciliated protozoa (i.e., ciliates), as well as the composition of the ciliates, in the rumen and reticulum of buffaloes in two feeding systems (grazing and feedlot with roughage added concentrate).

## Methods

Seventeen buffalo (*Bubalus bubalis*), 11 castrated males and six females, all Mediterranean breed, aged 23–26 months with live weight 469–562 kg, were fed two different diets. The animals were obtained from and housed on the Campus of Pirassununga of the University of Sao Paulo, Sao Paulo State, Brazil, and were cared for according to guidelines approved by the Comissao de Etica no Uso de Animais (CEUA) da Faculdade de Zootecnia e Engenharia de Alimentos da Universidade de Sao Paulo.

Twelve animals (six males and six females) were maintained on *Brachiaria brizantha* for 12 months. All 12 animals were slaughtered approximately 12 h after fasting. The remaining five animals, all males, were maintained in a feedlot for an additional 21 days where they received a diet consisting of 45 % silage corn and 55 % concentrate. The concentrate ration was composed of the following ingredients: 42.9 % corn grain ground, 30.0 % wheat meal, 23.0 % soybean seed roast, 1.9 % limestone, 0.2 % bicalcium phosphate, 2 % mineral salt. The composition of crude protein and neutral detergent fibre were as follows: concentrate [224 and 182 g/kg dry matter (DM)], *B. brizantha* (67 and 770 g/kg DM) and corn silage (72 and 440 g/kg DM).

Samples, consisting of liquid and fine particles, from five different regions within the rumen and five different regions within the reticulum were collected from each animal immediately after slaughter and the samples were pooled (by animal) to form a single sample (approximately 500 mL) from each of the two compartments of the stomach. After mixing the sample, 10 mL of rumen contents (solid and liquid) were preserved with a 50 % solution of formalin for later analysis of differential counts of ciliated protozoa. Another 4 mL sample of ruminal contents was stored in bottles containing 13 mL of 95 % ethanol (final solution of 77.6 % ethanol) and a 1.5 mL aliquots were transported to the Rumen Microbiology Laboratory at CSIRO Livestock Industries in Brisbane, Australia for DNA extraction, PCR amplification, and real-time PCR analysis of bacteria and total methanogenic archaea.

Identification and counting of the rumen and reticulum ciliate protozoa were developed in the Laboratory of Ruminal Metabolism of FZEA/USP, Brazil, using technique of counting individual cells by light microscopy [10]. Samples were mixed and a 1 mL aliquot was placed in a test tube, using a wide-bore pipet. The samples were stained with two drops of Brilliant Green overnight. An initial dilution was made with 9 mL of glycerin (30 % v/v) for a final dilution of 1:20. Further dilutions of 1:100 or 1:120 were made according to the concentration of ciliates in the sample observed in a Sedgewick–Rafter chamber with magnification of 100×. Counts were made using a counting grid, measuring 0.5 mm, located inside the eyepiece of the microscope. The ciliates were counted inside 100 grids along the total chamber (50 grids in the front side and 50 grids in the reverse side). Ciliates belonging to the subfamily Diplodiniinae (family Ophryoscolecidae) (e.g. *Diplodinium*, *Eudiplodinium*, *Ostracodinium*, *Metadinium*, *Enoploplastron* and *Polyplastron*) were counted together.

For each microbial group (bacteria, methanogenic archaea, and ciliate protozoa), extracted DNA from the rumen contents of water buffalo were analyzed in triplicate a using the real time PCR protocol [11]. Briefly, real-time PCR amplifications were carried out with the Bio-Rad Icycler in a 25 μL volume containing the following reagents: 12.5 μL SYBR green mix (QuantiTect™ SYBR^®^ Green PCR, Qiagen), 400 nM of each primer (Table 1) and 1.0 μL template DNA (10 ng). Real-time PCR amplification was initiated by a hot start at 95 °C for 15 min, followed by 40 cycles of 95 °C for 30 s, 60 °C for 30 s, and 72 °C for 60 s.

**Table 1:** Concentration of microorganisms into rumen (Ru) and reticulum (Re) in buffalo on grazing (G) and feedlot (F)

Microorganism	Rumen		Reticulum		p value			
					Ru	Re	Grazing	Feedlot
	G	F	G	F	G × F	G × F	Ru × Re	Ru × Re
*Entodinium* ^a^	2.56	1.34	2.23	1.32	0.0020	0.0820	0.0796	0.9747
Diplodiniinae^a^	2.34	0.29	1.92	0.30	0.0001	0.0001	0.1348	0.9213
*Epidinium* ^a^	0.09	0.00	0.21	0.01	0.0120	0.0001	0.1442	0.3466
*Isotricha* ^a^	0.02	0.00	0.06	0.00	0.1893	0.1189	0.0024	–
*Dasytricha* ^a^	0.20	0.00	0.95	0.00	0.0005	0.0071	0.0336	–
Total^a^	5.21	1.63	5.37	1.63	0.0001	0.0002	0.8766	1.0000
Bacteria^b^	3.89	1.30	1.20	0.71	0.2839	0.6490	0.2538	0.4071
Archaea^c^	0.66	2.40	6.00	2.32	0.1651	0.4936	0.3166	0.9624
%Archaea:bacteria	1.85	0.17	3.24	4.98				

^a^Number × 10^5^/mL

^b^Number × 10^11^/mL

^c^Number × 10^9^/mL

The external standards for rumen protozoa real-time PCR were as described and validated by Sylvester et al. [12] and ranged from 2.89 × 10^2^ to 2.89 × 10^6^ cells. The primers P-SSU-316f and P-SSU-539r were used in real-time PCR to target the 18S rRNA gene of ciliate protozoa [12]. The external standards for bacteria were described, validated and used a 6 log dilution series with the bacterial 16S rRNA gene primers 1114F and 1275R [13]. The external standards for methanogens were prepared using a mixture of pure cultures of *Methanobrevibacter ruminantium* M1^T^ and *Methanobrevibacter smithii* PS^T^, and ranged from 1.0 × 10^3^ to 1.0 × 10^8^ cells. Real-time PCR for methanogens was achieved using the primer pairs, qmcrA-F and qmcrA-R, to specifically target the methyl-coenzyme M reductase subunit [14].

Fluorescence was acquired during extension using an excitation wavelength of 470 nm, and emission detection at 530 nm. A final melting curve analysis was carried out by continuously monitoring fluorescence between 60C and 95 °C with 0.5 °C increments every 10 s. Threshold cycles were calculated automatically by the Icycler software (version 3.5). PCR efficiency for each extract was calculated from the logarithmic portion of the sigmoid shaped curve in real-time PCR reactions according to the methods described by Liu and Saint [15]. All data were statistically analysed according to random design using one-way ANOVA of GLM procedure of Statistica software [16].

## Results and discussion

No significant differences were observed between males and females. There were significant differences in the ciliate community between two feeding systems within the same gastric chamber, except for *Isotricha* (rumen or reticulum) and *Entodinium* in the reticulum (Table 1). The average values of different groups and of the total ciliates were higher in grazing buffalo than those in the feedlot on a concentrated diet.

There were significant interactions between location in the gastrointestinal tract (rumen vs reticulum) and types of diets (grazing vs feedlot) in the composition of ciliates (Table 2). Grazing buffalo showed a higher proportion of ciliates belonging to the subfamily Diplodiniinae, both in the rumen and reticulum as compared to feedlot diet, except for the *Isotricha*. No differences were observed in the value of percentages between rumen and reticulum (p > 0.05) in ciliates belonging to the subfamily Diplodiniinae. Species of *Isotricha* and *Dasytricha* (order Vestibuliferida; i.e., vestibuliferids) were denser in the reticulum than in the rumen in grazing buffalo and were not identified in feedlot animals.

**Table 2:** Composition of the protozoa community into rumen (Ru) and reticulum (Re) in buffalo on grazing (G) and feedlot (F)

Protozoa	Rumen (%)		Reticulum (%)		p value			
					Ru	Re	Pasture	Feedlot
	Grazing	Feedlot	Grazing	Feedlot	G × F	G × F	Ru × Re	Ru × Re
*Entodinium* ^a^	49.6	81.0	40.2	78.6	0.0001	0.0001	0.0009	0.5540
Diplodiniinae^a^	44.6	18.9	38.9	21.1	0.0001	0.0040	0.1264	0.6006
*Epidinium* ^a^	1.66	0.0	3.64	0.24	0.0069	0.0003	0.0020	0.3466
*Isotricha* ^a^	0.32	0.0	1.48	0.0	0.2163	0.1083	0.0549	–
*Dasytricha* ^a^	3.79	0.0	15.78	0.0	0.0003	0.0065	0.0012	–

^a^% of total of protozoa per mL of rumen fluid

There were no significant differences in the total concentrations of bacteria/mL and archaea between rumen and reticulum, and between pasture and feedlots, or interactions between variables. However, the largest density of bacteria and smallest density of archaea was observed in the rumen of grazing animals.

In general, the concentration of protozoa belonging to the genus *Entodinium* varied between 80 and 99 % for most domestic ruminant species, even those exclusively on forage diets [10]. However, our findings confirmed the existence of high concentration and composition of protozoa ciliates belonging to subfamily Diplodiniinae compared to protozoa of the genus *Entodinium* in grazing buffalo [17–19]. However, this did not occur in the feedlot animals, indicating that feeding with soluble carbohydrate favors the growth of *Entodinium*. Our current findings also showed that there is an indicative ecological niche of the vestibuliferids, *Isotricha* and *Dasytricha*, with a predominance in reticulum [20].

There is an important symbiotic association between methanogenic archaea and protozoa in the rumen [4]. Rumen protozoa are associated with high H_2_ production which is utilized by the methanogens associated either on the outside of or inside the protozoa [21, 22]. In the present study, methanogens appear to be associated with the vestibuliferid ciliates, *Isotricha and Dasytricha*, in the reticulum in grazing animals.

The methanogenic community in the rumen is small in proportion to the total density of bacteria ranging from 0.3 to 3.3 % [9]. In the case of concentrate diet used in feedlot, there were no vestibuliferid ciliates, and no differences between the rumen and reticulum, but the percentage of methanogens in relation to total bacteria was much higher in the reticulum (4.98 %) than in the rumen (0.17 %), indicating a possible selection acetate-producing bacteria that release major production of H_2_, thereby favoring the growth of archaea in the reticulum since in the acetate formation for each glucose fermentation there is a net balance of eight hydrogen atoms free [1].

The energy concentration and the dietary protein, as well as the carbohydrate and nitrogen sources have key roles in the concentration and composition of the microbiota in the rumen-reticulum [23]. Carbohydrate source (cassava chip and rice bran) did not affect rumen bacterial concentration in swamp buffalo, whereas cottonseed meal had a negative influence [24]. Also, replacing a rich diet of concentrate for an exclusive roughage diet increased cellulolytic bacteria in the swamp buffalo in diet with urea-treated rice straw with the highest concentration observed for *Fibrobacter succinogenes* [5].

## Conclusions

Considering the great diversity of microorganisms in the rumen with wide variation between ruminant animals distributed in different geographical regions in the world, our data showed differences in the microbial community of the rumen and reticulum between grazing and feedlot feeding systems demonstrating relevant changes in the microorganism:host relationship existing on rumen-reticulum ecosystem.

## Abbreviations

DM: dry matter
PCR: polymerase chain reaction
